# Analysis of Different Types of Interferon-Associated Retinopathy in Patients with Chronic Hepatitis C Virus Infection Treated with Pegylated Interferon Plus Ribavirin

**DOI:** 10.3390/v13030475

**Published:** 2021-03-14

**Authors:** Chia-Min Wu, Fu-Hsiung Su, Chih-Hsin Muo, Jou-Chen Huang, Meei-Maan Wu, Chih-Ching Yeh

**Affiliations:** 1 Department of Ophthalmology, Shuang Ho Hospital, Taipei Medical University, New Taipei City 235, Taiwan; windex034@gmail.com; 2 School of Public Health, College of Public Health, Taipei Medical University, Taipei 110, Taiwan; williamsufh1@yahoo.com.tw; 3 Department of Family Medicine, Cardinal Tien Hospital, Fu Jen Catholic University, New Taipei City 231, Taiwan; 4 School of Medicine, College of Medicine, Fu Jen Catholic University, New Taipei City 242, Taiwan; 5 Management Office for Health Data, China Medical University Hospital, Taichung 404, Taiwan; b8507006@gmail.com; 6 Department of Ophthalmology, Taipei Medical University Hospital, Taipei 110, Taiwan; roro691213@gmail.com; 7 Department of Public Health, School of Medicine, College of Medicine, Taipei Medical University, Taipei 110, Taiwan; 8 Master Program in Applied Molecular Epidemiology, College of Public Health, Taipei Medical University, Taipei 110, Taiwan; 9 Department of Public Health, College of Public Health, China Medical University, Taichung 404, Taiwan; 10 Cancer Center, Wan Fang Hospital, Taipei Medical University, Taipei 116, Taiwan

**Keywords:** hepatitis C virus, interferon-associated retinopathy, retinal hemorrhage, retrospective cohort study

## Abstract

This retrospective cohort study aims to investigate interferon (IFN)-associated retinopathy incidence in patients with chronic hepatitis C virus (HCV) infection treated with pegylated interferon (PegIFN) plus ribavirin (RBV). We selected 1688 patients undergoing PegIFN/RBV therapy for HCV (HCV-treated cohort), 3376 patients not receiving HCV treatment (HCV-untreated cohort) and 16,880 controls without HCV (non-HCV cohort) from the Taiwan Longitudinal Health Insurance Database. The patients were frequency-matched by age, sex, and index date at a 1:2:10 ratio, and followed up until the end of 2013. Cox proportional hazard regression models were used to compare the incidences of any retinal vascular events, including subtypes, among the three cohorts. Compared with the non-HCV cohort, the HCV-treated cohort had a significantly increased risk of retinopathy (hazard ratio (HR) = 4.98, 95% confidence interval (CI): 2.02–12.3). The risk was particularly prominent for retinal hemorrhage (HR = 12.7, 95% CI: 3.78–42.9). When the HCV-untreated cohort was used as the reference, the aforementioned HRs increased to 9.02 (95% CI: 3.04–26.8) and 32.3 (95% CI: 3.94–265), respectively. This study suggested that PegIFN/RBV therapy significantly increased the risk of retinal hemorrhage but not retinal vascular occlusions in the HCV-treated cohort.

## 1. Introduction

Hepatitis C virus (HCV) infection is a global health threat; in 2015, approximately 180 million people worldwide had chronic HCV infection [1]. In addition to liver diseases, chronic HCV infection is associated with several extrahepatic manifestations, including mixed cryoglobulinemia, type 2 diabetes mellitus, chronic kidney disease, lymphoma, lichen planus, Sjogren’s syndrome, porphyria cutanea tarda, depression, cardiovascular disease, and rheumatoid arthritis [2]. Epidemiological evidence supports an association of chronic HCV infection with ocular manifestations, such as dry eye syndrome and ischemic retinopathy [3]. Ischemic retinopathy may be a side effect of interferon therapy or a manifestation of the systemic vasculitis induced by the infection.

Interferons (IFNs) have been used effectively for chronic HCV infection treatment; however, the multiple injections required per week is a burden. Pegylated interferon (PegIFN), a chemically modified IFN form, prolongs the drug half-life and reduces the dosing frequency [4]. PegIFN plus ribavirin (RBV) has been frequently used to treat patients with HCV infection in the one or two decades since the response-guided therapy reimbursement guidelines were promulgated in Taiwan [5,6,7]. IFN- and PegIFN-based therapy may induce retinopathy [8], the incidence of which varies from 16 to 86% [9]. In a review, the pooled incidence of retinopathy in 10 studies that used PegIFN only was 20.9% [10]. Although sporadic severe events, such as ischemic retinopathy or optic neuropathy, that eventually affect vision have been reported, the most commonly encountered types of IFN-associated retinopathy were cotton wool spots and retinal hemorrhage, which are typically asymptomatic and reversible [11,12,13,14,15]. However, these ocular syndromes were reported in small case series and individual patients. In a retrospective cohort study involving 4736 patients from the Taiwan National Health Insurance (NHI) Research Database, Lai et al. (2018) indicated that the risk of retinopathy was 1.53 times higher in patients receiving interferon–ribavirin therapy for HCV infection than in patients with HCV not receiving the therapy (95% confidence interval (CI), 1.14–2.06; *p* = 0.0048) [16]. Nevertheless, the study did not adjust for all potential confounding factors or evaluate retinopathy subtypes. Furthermore, studies have suggested that patients with HCV and comorbidities such as diabetes and hypertension have an increased risk of ocular complications, but the results have been inconsistent [16,17].

Because retinopathy might be caused by HCV infection or induced by therapy, we compared patients receiving treatment for HCV (HCV-treated cohort), those not receiving treatments for HCV (HCV-untreated cohort), and those without HCV (non-HCV cohort). We analyzed the incidence rates of any retinal vascular events among the three cohorts as well as PegIFN/RBV-associated retinopathy between the HCV-untreated and HCV-treated cohorts, using a nationwide, large-scale, retrospective cohort study design.

## 2. Materials and Methods

### 2.1. Database

In 1995, Taiwan established its single-payer NHI programme to provide affordable healthcare for the entire population. As of 2009, this compulsory-enrolment program covered 99.9% of Taiwan’s population [18].

We retrieved the data of sampled participants from the Taiwan Longitudinal Health Insurance Database (LHID2000), which was created and is maintained by the Taiwan National Health Research Institute (NHRI). The LHID2000 includes all medical claims and registration files for 1,000,000 beneficiaries in the Taiwan NHI program. A set of 1,000,000 patient data records in the LHID2000 were randomly selected from all enrolees listed in the 2000 Registry of Beneficiaries (N = 23.72 million). The Taiwan NHRI validated the representativeness of the LHID2000 relative to the entire population of NHI enrolees in terms of age, sex, and monthly income distributions. This study was approved by the Institutional Review Board and the Hospital Research Ethics Committee of China Medical University (Institutional Review Board approval number: CMU-REC-101-012).

### 2.2. Study Sample

**Figure 1:**
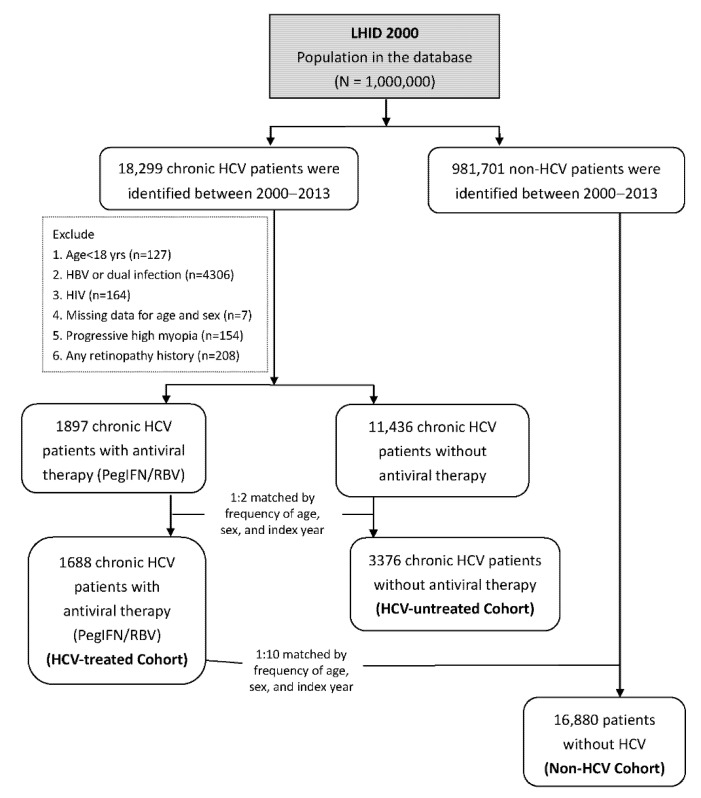
Flow chart of patient enrolment.

Figure 1 presents the flowchart for study patient recruitment from the LHID2000 database. We first identified 18,299 patients who had received a first diagnosis of chronic HCV infection (International Classification of Diseases, Ninth Revision, Clinical Modification (ICD-9-CM) codes 070.41, 070.44, 070.51, 070.54,070.70,070.71 and V02.62) between January 1, 2000, and December 31, 2013. Patients who received a second diagnosis of HCV infection 6 months after being diagnosed as having acute or unspecified HCV were classified as having chronic HCV. We selected the date of antiviral therapy as the index date. We also excluded patients aged < 18 years (*n* = 127) and those with chronic hepatitis B virus infection (ICD-9-CM codes 070.2, 070.3, and V02.61; *n* = 4306), human immunodeficiency virus infection (ICD-9-CM codes 042, 043, 044, V08, and 795.8, *n* = 164), missing data for age or sex (*n* = 7), progressive high myopia (ICD-9-CM code 360.21, *n* = 154), and any retinal vascular events (ICD9-CM codes 362.35, 362.36, and 362.37 for retinal venous occlusion; 362.31, 362.32, 362.33, and 362.34 for retinal arterial occlusion; 362.81 for retinal hemorrhage; and 362.30, 362.82, and 362.83 for unspecified types; *n* = 208) prior to the index date. After frequency matching by age, sex, and year of index date at a 1:2 ratio, 1688 patients with HCV infection who received PegIFN/RBV treatment were included in the HCV-treated cohort and 3376 patients with HCV infection without PegIFN/RBV treatment were included in the HCV-untreated cohort. For the non-HCV cohort, we retrieved data from the remainder of the NHI enrolees in the LHID2000 and applied the same exclusion criteria as those used for the HCV cohort. We performed frequency matching by age, sex, and year of the index date at a 1:10 ratio (HCV-treated:non-HCV = 1:10) to select the comparison cohort participants. Consequently, 16,880 patients were included in the non-HCV cohort. Each patient was individually followed up for 2 years after their index date to identify those who developed any retinopathy events. The date of first diagnosis of any retinopathy type during the follow-up period or by the end of the study was considered the study endpoint.

### 2.3. Statistical Analysis

All tests were two-sided, and the significance level was set to 0.05. The dataset did not offer information regarding the laterality of the cases. The primary aim was to examine whether the cumulative incidence rates of retinopathy (including retinal venous occlusion, retinal arterial occlusion, retinal hemorrhage, and other unspecified retinal event subtypes) differ among the non-HCV, HCV-untreated, and HCV-treated cohorts by using Kaplan–Meier curves and a log-rank test. We also assessed whether PegIFN/RBV use for ≥24 weeks (minimum required period for efficient treatment) increases retinopathy risk. In addition, the side effects of anemia (ICD9-CM codes 280–285) and thrombocytopenia (ICD9-CM codes 287.4 and 287.5) induced by PegIFN/RBV treatment were evaluated. The Lunn–McNeil approach was used to modify the Cox proportional hazards models to consider competing risks [19]. The extended Cox proportional hazard models with the competing risk of death were used to evaluate the relative risk of retinal vascular events among patients in the three cohorts. Hazard ratios (HRs) and 95% CIs were estimated in the models. The potential covariates included in the multivariate models were age, sex, socioeconomic factors (i.e., occupation category, urbanization level, residence area, and monthly income), comorbidities (i.e., myocardial infarction (ICD-9-CM codes 410–414), cerebrovascular disease (ICD-9-CM codes 430–438), chronic pulmonary disease (ICD-9-CM codes 490–505 and 506.4), diabetes mellitus (DM; ICD-9-CM code 250), chronic renal disease (ICD-9-CM codes 582–583.7, 584–586, 588), hypertension (ICD-9-CM codes 401–405), hyperlipidaemia (ICD-9-CM codes 272.0–272.2), cataract (ICD-9-CM codes 366.0, 366.1, and 366.9), diabetic retinopathy (ICD-9-CM codes 362.01–362.07), liver cirrhosis (ICD-9-CM codes 571), obesity (ICD-9-CM code 278) and anemia (ICD-9-CM codes 280–285)), and the use of hydroxyl-methyl-glutaryl coenzyme A (HMG Co-A) reductase inhibitor (statin), acetylsalicylic acid (aspirin), and other nonsteroidal anti-inflammatory drugs (NSAIDs). Socioeconomic status was determined according to baseline data at the index date, and comorbidities were recorded during the 12 months before the index date.

The study drugs were identified according to the anatomical therapeutic chemical (ATC) classification system codes from the dataset. PegIFN/RBV usage was defined as ATC codes L03AB04, L03AB05, L03AB09, L03AB10, L03AB11, L03AB60, and L03AB61. The types of statin used included lovastatin (C10AA02), pravastatin (C10AA03), fluvastatin (C10AA04), simvastatin (C10AA01), atorvastatin (C10AA05), and rosuvastatin (C10AA07). Statin users were defined as patients who had a cumulative defined daily dose (cDDD) of >28 in the year preceding the index date. Aspirin (B01AC) and other NSAID (M01AH, M01AA, M01AB, M01AC, M01AE, M01AG, and M01AX) users were defined as patients who had prescriptions for relevant medications for at least 3 consecutive months in the year preceding the index date.

## 3. Results

### 3.1. General Characteristics of Patients

Table 1 presents the demographic characteristics and comorbidities of the study participants. Enrolees in the HCV cohort were more likely to have a blue-collar occupation, live in a rural area or in southern Taiwan, and have a lower income than those in the non-HCV cohort were. The prevalence of myocardial infarction, cerebrovascular disease, chronic pulmonary disease, DM, renal disease, hypertension, hyperlipidaemia, cataract, diabetic retinopathy, liver cirrhosis, and anemia was significantly higher in the HCV cohort than in the non-HCV cohort. Participants in the HCV cohort were less likely to use statins than their non-HCV counterparts were.

### 3.2. Survival Analysis for Interferon-Associated Retinopathy among Study Cohorts

**Figure 2:**
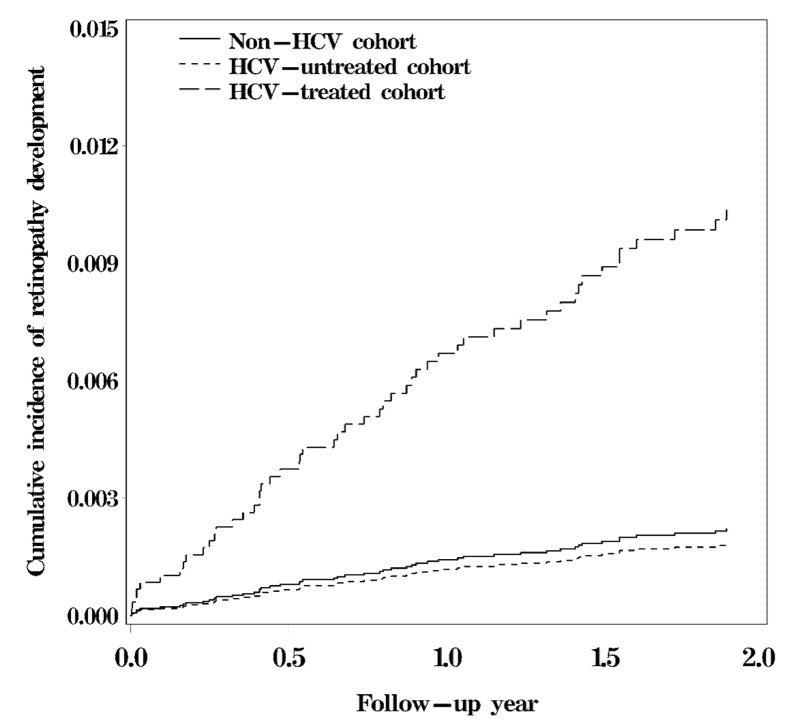
Cumulative risk of retinopathy among the HCV-treated, HCV-untreated, and non-HCV cohorts (log-rank test, *p* < 0.0001; HCV-untreated cohort vs. non-HCV cohort, *p* = 0.70; HCV-treated cohort vs. non-HCV cohort, *p* < 0.0001; HCV-treated cohort vs. HCV-untreated cohort, *p* = 0.0008).

The survival curve revealed a significant difference in the cumulative incidence of any retinopathy among the HCV-treated, HCV-untreated, and non-HCV cohorts (log-rank test, *p* < 0.05; Figure 2). The 2-year cumulative incidence of any retinopathy was approximately 0.89% in the HCV-treated cohort, 0.15% in the HCV-untreated cohort, and 0.19% in the non-HCV cohort.

### 3.3. Risk of Interferon-Associated Retinopathy among Study Cohorts

The incidence rates and adjusted HRs of all retinopathy events for the three cohorts are provided in Table 2. The retinal hemorrhage subtype accounted for 73% (11/15) of IFN-associated retinopathy cases in the HCV-treated cohort. Retinal hemorrhage accounted for 38% (12/32) and 20% (1/5) of any retinopathy cases in the non-HCV and HCV-untreated cohorts, respectively (Table 2). Compared with the non-HCV cohort, the adjusted HR (95% CI) for any retinopathy in the HCV-treated cohort was 4.98 (2.02–12.3). This significantly increased risk of retinopathy was particularly enhanced for the retinal hemorrhage and retinal arterial occlusion subtypes, with HRs (95% CIs) of 12.7 (3.78–42.9) and 90.6 (4.41–1865), respectively. However, no significant relationship was identified between patients in the HCV-untreated and non-HCV cohorts. When the HCV-untreated cohort was used as the reference, the HRs (95% CIs) for any retinopathy and retinal hemorrhage in the HCV-treated cohort were 9.02 (3.04–26.8) and 32.2 (3.94–265), respectively.

Table 3 indicates that the risk of any retinopathy events was significantly associated with PegIFN/RBV therapy, regardless of whether patients received an efficient HCV treatment. Patients receiving HCV treatment for ≥24 weeks exhibited a higher risk of any retinopathy (HR = 8.18, 95% CI: 2.58–25.9) and retinal hemorrhage (HR = 36.2, 95% CI: 4.19–313) compared with the HCV-untreated cohort. We also observed that patients receiving PegIFN/RBV treatment for <24 weeks had an increased risk of any retinopathy (HR = 11.3, 95% CI: 3.00–42.8) and retinal hemorrhage (HR = 22.0, 95% CI: 1.91–254).

### 3.4. Risk of Side Effects Induced by PegIFN/RBV Treatment among Study Cohorts

We also explored the side effects of anemia and thrombocytopenia induced by the PegIFN/RBV treatment before retinopathy development (Table 4). The HCV-treated cohort had a significantly increased risk of anemia compared with the non-HCV cohort (HR = 8.10, 95% CI: 5.90–11.1) and the HCV-untreated cohort (HR = 4.89, 95% CI 3.66–6.55). The HCV-untreated cohort also exhibited a higher risk of anemia (HR = 1.53, 95% CI: 1.10–2.13). A significantly increased risk of thrombocytopenia was observed in the HCV-treated (HR = 4.11, 95% CI: 2.22–7.61) and HCV-untreated (HR = 3.75, 95% CI: 2.15–6.54) cohorts compared with the non-HCV cohort. However, the difference between the HCV-treated and -untreated cohorts was nonsignificant.

## 4. Discussion

In our study, PegIFN/RBV-associated retinopathy was defined as any retinopathy, including retinal venous occlusions, retinal arterial occlusions, retinal hemorrhage, and other unspecified retinopathy. The HCV-treated cohort had a significantly increased risk of any retinopathy (HR = 4.98) and the retinal hemorrhage subtype (HR = 12.7) than non-HCV patients (Table 2). We observed a comparable incidence of more advanced events, such as retinal venous or arterial occlusions, in the non-HCV, HCV-untreated, and HCV-treated cohorts, and a significantly higher incidence of the less advanced retinal hemorrhage subtype in the HCV-treated cohort. Our findings of the equal incidence of advanced retinopathy events among the non-HCV, HCV-untreated, and HCV-treated cohorts are consistent with a previous study that indicated that the incidence of severe retinopathy was low [20], suggesting that routine examination for retinopathy is unnecessary. Because most typical retinal findings of IFN-associated retinopathy, including cotton wool spots and retinal hemorrhage, are benign and reversible, referring only patients with subjective vision changes to ophthalmologists for fundus examination is reasonable, instead of performing routine fundus examination during the PegIFN/RBV treatment course to prevent severe ocular ischemic events, just as patients without HCV would be evaluated.

We did not identify differences in retinopathy incidence between the treatment failure (treatment for <24 weeks) and treatment success (treatment for ≥24 weeks) cohorts. Our real-world data confirmed that both successful and failed PegIFN/RBV treatment significantly increased the risk of retinopathy. Although a vast majority of IFN-associated retinopathy occurred 2–24 weeks posttreatment, as revealed by previous studies, a considerable cumulative number of cases were detected after 24 weeks based on our results [11,13,14]. A previous study revealed that some parameters of retinal circulation (including retinal blood flow, blood velocity, and retinal wall shear rate) remained increased 24 weeks after IFN treatment compared with baseline data [21]. Some studies with limited follow-up periods have revealed that although most IFN-associated retinopathy events occur early in treatment, the number of cases still increases later in IFN treatment. Accordingly, we recommend that patients’ subjective vision changes be monitored throughout IFN treatment.

In studies on IFN- or PegIFN-based therapies, the most common subtypes of IFN-associated retinopathy have been cotton wool spots and retinal haemorrhage [11,13,14]. In the present study, the retinal hemorrhage subtype was the most common, accounting for 73% of cases in the HCV-treated cohort and 38% and 20% of cases in the non-HCV and HCV-untreated cohorts, respectively. Cotton wool spots are ambiguously attributed to ICD-9-CM code 362.83 (retinal edema), which may lead to their underestimation. However, we presumed that selection bias regarding cotton wool spots among the non-HCV, HCV-untreated, and HCV-treated cohorts was undifferential. Retinal hemorrhage is also a typical finding of anemia or thrombocytopenia in retinopathy. IFN-based therapies and RBV have dose-dependent side effects of anemia and thrombocytopenia [22,23,24,25,26,27,28]. Our study revealed a higher incidence of anemia and thrombocytopenia in the HCV-treated cohort. Anemia and thrombocytopenia were reported to be associated with retinal hemorrhage [29]. Anemia-related tissue hypoxia may cause vasodilatation and abnormal vessel leakage, leading to fundus abnormalities [29,30,31,32]. We presumed that diagnoses of IFN-associated retinopathy would inevitably include anemic or thrombocytopenic retinopathy, induced by PegIFN or RBV in a real-world data set. However, in our study, the causal relation between IFN-associated retinopathy and hematological conditions was difficult to clarify because of the small number of cases for comparison. A possible explanation may be that most patients with retinal hemorrhage caused by hematological disorders are asymptomatic and will not seek medical care.

This study has two main strengths. First, we included both non-HCV and HCV-untreated cohorts for comparison. Therefore, we could distinguish normal and abnormal events in a specific cohort of patients with HCV receiving PegIFN/RBV treatment. In most previous case series, a lack of data from a comparison cohort has resulted in inaccurate interpretations of PegIFN/RBV-associated retinopathy. Second, this was a nationwide population-based study, with a large sample size enrolled from the NHIRD.

This study has several limitations. First, we acquired diagnoses of retinopathy by using the ICD-9-CM codes in claims records, instead of retinal images of every case. Moreover, our results were acquired in a retrospective and observational manner, rather than in an interventional study with more rigorous ophthalmic follow-up. These limitations may have resulted in the underestimation of the true incidence of PegIFN/RBV-associated retinopathy, particularly that of asymptomatic retinal hemorrhage and cotton wool spots. Second, we could not identify the resolution of PegIFN/RBV-associated retinopathy or visual outcomes in the database. Third, because not every patient with anemia or thrombocytopenia receives routine fundus examination, some cases of asymptomatic anemia or thrombocytopenia may have been missed in the analysis.

## 5. Conclusions

In conclusion, we identified a significantly increased risk of retinal hemorrhage, but a similar risk of retinal vascular occlusions, in the HCV cohort treated with PegIFN/RBV compared with the HCV-untreated cohort. Because most cases of retinal hemorrhage are considered benign and reversible, and advanced events are infrequent, we suggest that routine fundus examination is not required except in patients with complaints of subjective vision changes.

## Figures and Tables

**Table 1:** Sociodemographic characteristics and comorbidities of the non-hepatitis C virus (HCV), HCV-untreated, and HCV-treated cohorts.

Parameters	Non-HCV (*n* = 16,880)	HCV-Untreated (*n* = 3376)	HCV-Treated (*n* = 1688)	*p* Value
Age, years	51.5 (12.0)	51.6 (12.0)	51.4 (11.9)	0.88
Male sex, *n* (%)	9130 (54.1)	1826 (54.1)	913 (54.1)	>0.99
Occupation				<0.0001
White-collar	9545 (56.6)	1439 (42.6)	709 (42.0)	
Blue-collar	6060 (35.9)	1646 (48.8)	873 (51.7)	
Other	1275 (7.55)	291 (8.62)	106 (6.28)	
Urbanisation				<0.0001
Urban	5379 (31.9)	753 (22.3)	362 (21.5)	
Suburban	8074 (47.8)	1572 (46.6)	794 (47.0)	
Rural	3427 (20.3)	1051 (31.1)	532 (31.5)	
Geographic region				<0.0001
Northern	8015 (47.5)	1011 (30.0)	466 (27.6)	
Central	3286 (19.5)	735 (21.8)	366 (21.7)	
Southern	4782 (28.3)	1429 (42.3)	764 (45.3)	
Eastern and outlying islands	797 (4.72)	201 (5.95)	92 (5.45)	
Monthly Income (NT$)				<0.0001
<15,840	5845 (34.6)	1008 (29.9)	437 (25.9)	
15,840–24,999	6431 (38.1)	1689 (50.0)	884 (52.4)	
≥25,000	4604 (27.3)	679 (20.1)	367 (21.7)	
Comorbidities				
Myocardial infarction	2092 (12.4)	780 (23.1)	313 (18.5)	<0.0001
Cerebrovascular disease	1182 (7.00)	454 (13.5)	184 (10.9)	<0.0001
Chronic pulmonary disease	4025 (23.8)	1204 (35.7)	623 (36.9)	<0.0001
Diabetes mellitus	2328 (13.8)	960 (28.4)	475 (28.1)	<0.0001
Renal disease	489 (2.90)	348 (10.3)	96 (5.69)	<0.0001
Hypertension	4830 (28.6)	1503 (44.5)	745 (44.1)	<0.0001
Hyperlipidaemia	1916 (11.4)	548 (16.2)	240 (14.2)	0.002
Cataract	1572 (9.31)	642 (19.0)	323 (19.1)	<0.0001
Diabetic retinopathy	242 (1.43)	112 (3.32)	39 (2.31)	<0.0001
Liver cirrhosis	2697 (16.0)	2448 (72.5)	1537 (91.1)	<0.0001
Obesity	362 (2.14)	83 (2.46)	45 (2.67)	0.24
Anemia	1388 (8.22)	595 (17.6)	249 (14.8)	<0.0001
Medications				
Statin	1182 (7.00)	212 (6.28)	73 (4.32)	<0.0001
Aspirin	18 (0.11)	8 (0.24)	2 (0.12)	0.15
Nonsteroidal anti-inflammatory drugs	51 (0.30)	14 (0.41)	10 (0.59)	0.11

Data are expressed as mean (standard deviation) or number (percentage).

**Table 2:** Incidence rates and hazard ratios for the risk of retinopathy among the non-HCV, HCV-untreated, and HCV-treated cohorts.

Retinopathy		Non-HCV	HCV-Untreated	*p* Value	HCV-Treated	*p* Value
Any retinopathy	Cases	32	5		15	
	Person-years	27,789	5032		2756	
	Incidence (10^−3^)	1.15	0.99		5.44	
	HR ^a^ (95% CI)	1.00 (ref)	0.71 (0.24–2.14)	0.55	4.98 (2.02–12.3)	0.0005
			1.00 (ref)		9.02 (3.04–26.8)	<0.0001
Retinal venous occlusion	Cases	11	3		3	
	Person-years	27,789	5032		2756	
	Incidence (10^−3^)	0.40	0.60		1.09	
	HR ^a^ (95% CI)	1.00 (ref)	0.70 (0.15–3.21)	0.64	1.33 (0.29–6.18)	0.72
			1.00 (ref)		1.90 (0.40–9.00)	0.42
Retinal arterial occlusion	Cases	3	0		1	
	Person-years	27,789	5032		2756	
	Incidence (10^−3^)	0.11	0.00		0.36	
	HR ^a^ (95% CI)	1.00 (ref)	NA	0.71	90.6 (4.41–1865)	0.0004
			1.00 (ref)		NA	
Retinal hemorrhage	Cases	12	1		11	
	Person-years	27,789	5032		2756	
	Incidence (10^−3^)	0.43	0.20		3.99	
	HR ^a^ (95% CI)	1.00 (ref)	0.48 (0.07–3.40)	0.46	12.7 (3.78–42.9)	<0.0001
			1.00 (ref)		32.3 (3.94–265)	0.001
Unspecified	Cases	6	1		0	
	Person-years	27,789	5032		2756	
	Incidence (10^−3^)	0.22	0.20		0.00	
	HR ^a^ (95% CI)	1.00 (ref)	1.27 (0.03–47.3)	0.90	NA	
			1.00 (ref)		NA	

^a^ Adjusted for age, sex, occupation, urbanization level, geographical region, monthly income, myocardial infarction, cerebrovascular disease, chronic pulmonary disease, diabetes mellitus, renal disease, hypertension, hyperlipidaemia, cataract, diabetic retinopathy, liver cirrhosis, anemia, statin use, and competing mortality. Abbreviations: CI, confidence interval; HR, hazard ratio.

**Table 3:** Risk of retinopathy associated with anti-HCV therapy and treatment duration.

		HCV-Untreated	HCV Patients with Antiviral Therapy (PegIFN/RBV)			
Retinopathy			<24 Weeks	*p* Value	≥24 Weeks	*p* Value
Any retinopathy	Cases	5	5		10	
	Person-years	5032	562		2194	
	Incidence (10^−3^)	0.99	8.90		4.56	
	HR ^a^ (95% CI)	1.00 (ref)	11.3 (3.00–42.8)	0.0003	8.18 (2.58–25.9)	0.00004
Retinal venous occlusion	Cases	3	2		1	
	Person-years	5032	562		2194	
	Incidence (10^−3^)	0.60	3.56		0.46	
	HR ^a^ (95% CI)	1.00 (ref)	6.59 (0.94–46.2)	0.06	0.76 (0.11–5.35)	0.78
Retinal arterial occlusion	Cases	0	1		0	
	Person-years	5032	562		2194	
	Incidence (10^−3^)	0.00	1.78		0.00	
	HR ^a^ (95% CI)	1.00 (ref)	NA		NA	
Retinal hemorrhage	Cases	1	2		9	
	Person-years	5032	562		2194	
	Incidence (10^−3^)	0.20	3.56		4.10	
	HR ^a^ (95% CI)	1.00 (ref)	22.0 (1.91–254)	0.01	36.2 (4.19–313)	0.001
Unspecified	Cases	1	0		0	
	Person-years	5032	562		2194	
	Incidence (10^−3^)	0.20	0.00		0.00	
	HR ^a^ (95% CI)	1.00 (ref)	NA		NA	

^a^ Adjusted for age, sex, occupation, urbanization, geographical region, monthly income, myocardial infarction, cerebrovascular disease, chronic pulmonary disease, diabetes mellitus, renal disease, hypertension, hyperlipidemia, cataract, diabetic retinopathy, liver cirrhosis, anemia, statin, and competing mortality. Abbreviations: CI, confidence interval; HR, hazard ratio.

**Table 4:** Risk of anemia and thrombocytopenia among the non-HCV, HCV-untreated, and HCV-treated cohorts.

		Non-HCV	HCV-Untreated	*p* Value	HCV-Treated	*p* Value
Anemia	Cases	179	77		152	
	Person-years	27,740	5007		2667	
	Incidence (10^−3^)	6.45	15.38		56.99	
	HR ^a^ (95% CI)	1.00 (ref)	1.53 (1.10–2.13)	0.01	8.10 (5.90–11.1)	<0.0001
			1.00 (ref)		4.89 (3.66–6.55)	<0.0001
Thrombocytopenia	Cases	39	52		34	
	Person-years	27,789	5032		2756	
	Incidence (10^−3^)	1.40	10.33		12.34	
	HR ^a^ (95% CI)	1.00 (ref)	3.75 (2.15–6.54)	<0.0001	4.11 (2.22–7.61)	<0.0001
			1.00 (ref)		1.13 (0.71–1.80)	0.60

^a^ Adjusted for age, sex, occupation, urbanization, geographical region, monthly income, myocardial infarction, cerebrovascular disease, chronic pulmonary disease, diabetes mellitus, renal disease, hypertension, hyperlipidemia, cataract, diabetic retinopathy, liver cirrhosis, anemia, statin, and competing mortality. Abbreviations: CI, confidence interval; HR, hazard ratio.

## Data Availability

All data are available from the NHIRD of Taiwan (http://nhird.nhri.org.tw/en/index.htm) (accessed on 24 February 2021). Requests for data can be sent as a formal proposal to the NHIRD.
